# Noninvasive fetal antigen genotyping: Results from a survey on the status of clinical implementation

**DOI:** 10.1111/vox.70062

**Published:** 2025-06-09

**Authors:** Frederik Banch Clausen, Åsa Hellberg, Cécile Toly‐Ndour, Emilie Thorup Nielsen, Masja de Haas, The Noninvasive Fetal Antigen Genotyping Survey Group, Çiğdem Akalin Akkök, Aseel Alshamari, Ivana Babic, Tonje Espeland Bagås, Frédéric Bauer, Jakob T. Bay, Rhita Bennis, Gwen Clarke, Jean‐Marc Costa, Carlos Cotorruelo, Christoffer D. Dellgren, Andrea Doescher, Tadeja Dovc Drnovsek, Ducreux Stéphanie, Badrdine El Masmouhi, Willy Albert Flegel, Martine Florent, Gatouillat Grégory, Emmanuelle Guinchard, Sys Hasslund, Christine Henny, Kaimo Hirv, Camilla Holmertz, Jayne Houghton, Jauffret Vincent, Yanli Ji, Hou Jue, Elisa Kaivanto, Margaret Keller, Patrick SL Kwan, Annie Levy, Shuang Liang, Yew‐Wah Liew, Loosen Claire, Agnès Mailloux, Jean‐Marc Minon, Kenneth J. Moise, Núria Nogués, Magnus Nordström, Agnieszka Orzińska, Armin Pahl, Cecilia Pardi, Pascal Pedini, Helen Ryan, Janine‐Nicole Sorrentino, Rudi Steffensen, Ingvild H. Sørvoll, Jenny Bjork Thorsteinsdottir, Sandra Wienzek‐Lischka

**Affiliations:** ^1^ Laboratory of Blood Genetics, Department of Clinical Immunology Copenhagen University Hospital, Rigshospitalet Copenhagen Denmark; ^2^ cfDNA Subgroup from the International Society of Blood Transfusion (ISBT) Working Party on Red Cell Immunogenetics and Blood Group Terminology (RCIBGT) Amsterdam The Netherlands; ^3^ Department of Clinical Immunology and Transfusion Medicine Office of Medical Service, Region Skåne Lund Sweden; ^4^ Laboratory of the French National Reference Center in Perinatal Hemobiology, Assistance Publique des de Paris (AP‐HP) Paris France; ^5^ Center of Fetal Medicine, Department of Gynecology, Fertility and Pregnancy Copenhagen University Hospital, Rigshospitalet Copenhagen Denmark; ^6^ Department of Immunohaematology Diagnostic Services Sanquin Diagnostic Services and Sanquin Research Amsterdam The Netherlands; ^7^ Department of Haematology Leiden University Medical Center Leiden The Netherlands

**Keywords:** blood group, cell‐free fetal DNA, HDFN, prophylaxis, RhD

## Abstract

**Background and Objectives:**

Noninvasive fetal antigen genotyping can assist the management of immunized pregnant women, and fetal *RHD* genotyping can be used as a screening assay to guide restricted use of Rh prophylaxis. Based on a survey, we present an overview of the implementation of noninvasive fetal antigen genotyping into clinical practice.

**Materials and Methods:**

A survey was developed and sent out to the members of the International Society of Blood Transfusion (ISBT) working party on Red Cell Immunogenetics and Blood Group Terminology and to participants of the international noninvasive fetal *RHD* genotyping programme from the Danish Institute for External Quality Assurance for Laboratories in the health sector (DEKS). The survey contained four sections: fetal *RHD* screening, fetal *RHD* genotyping for RhD immunized women, fetal genotyping of other antigen targets than RhD and a section for collecting topics for future collaboration. The survey data were evaluated by the core author group.

**Results:**

Fifty‐two survey responders were from 22 countries. Fetal *RHD* screening was implemented by 73%, foremost using real‐time PCR with *in‐house* assays, primarily, or commercially available kits. Most laboratories use the same assay for RhD immunized women. Thirty‐eight percent of the responders test for other antigen targets than RhD, using either real‐time PCR, droplet digital PCR (ddPCR) or DNA sequencing. There was an interest in collaborations on topics across methodology, technology, strategy and health care regulations.

**Conclusion:**

In general, we found that noninvasive fetal blood group antigen genotyping is well implemented. However, our results are biased towards high‐income countries, Europe and laboratories already running noninvasive fetal antigen genotyping.

**Highlights**

- Noninvasive fetal antigen genotyping is used for predicting fetal blood groups during pregnancy.
- Fetal *RHD* screening can be used to guide targeted use of Rh prophylaxis.
- Noninvasive fetal antigen genotyping and fetal *RHD* screening are well integrated into clinical routine in many countries.

## INTRODUCTION

In the management of immunized pregnant women, noninvasive fetal antigen genotyping can be applied diagnostically for assessing the risk of haemolytic disease of the fetus and newborn [1, 2]. In addition, noninvasive fetal *RHD* genotyping can be used as a screening of non‐immunized RhD negative pregnant women to guide the use of targeted Rh immunoglobulin (RhIG) prophylaxis [3], restricting the prophylaxis only to those women who will benefit from it. Restricting Rh prophylaxis is highly important as the availability of RhIG is limited globally [4, 5]. There are still no monoclonal antibodies that have shown their effectiveness to prevent anti‐D immunization, with the exception of Rhoclone, which needs more extensive trials to establish its efficacy [6]. The use of RhIG also raises the infectious risks related to all blood‐derived products as well as ethical issues, including deliberate immunization, payment of the donors, production in a few numbers of countries—all issues warranting restricted use of RhIG. The American College of Obstetricians and Gynecologists (ACOG) has recently issued a practice advisory recommending using noninvasive fetal *RHD* genotyping to prioritize use of RhIG and conserve its supply if experiencing RhIG shortage [7, 8].

Noninvasive fetal antigen genotyping is based on the analysis of cell‐free fetal DNA (cffDNA) extracted from maternal plasma, allowing for a noninvasive and accurate prediction of a fetal antigen during pregnancy [9]. With its first application more than 20 years ago [10], noninvasive fetal antigen genotyping has become a standard routine service of transfusion medicine, foremost in high‐income countries, and nationwide fetal *RHD* screening programmes have been part of clinical routine in many countries in Europe for almost 15 years [3, 9, 11, 12, 13, 14].

At the International Society of Blood Transfusion (ISBT), the cell‐free DNA (cfDNA) Subgroup works to advance clinical applications of cffDNA testing in transfusion medicine, as a subgroup of the Red Cell Immunogenetics and Blood Group Terminology Working Party (RCIBGT WP). In this context, we aimed to assess the current level of implementation of noninvasive fetal antigen genotyping as well as to gain additional insight into the applied methodologies.

An overview of the implementation of noninvasive fetal antigen genotyping is important as this area is facing different challenges, including limited access to Rh prophylaxis, potential need for standardization, increased commercialized interest and, in Europe, increased regulations that may unnecessarily delay or hinder the prospect for implementation and continuation of well performing *in‐house* developed assays. Simultaneously, technological advancements have permitted easier analysis of immunized women, and in conjunction with a growing knowledge of, for example, the molecular background of blood groups, laboratories are interested in expanding diagnostic services for pregnant women. Identifying key differences, efficient strategies and addressing potential challenges may assist in the advancement forward.

## MATERIALS AND METHODS

A survey was developed to evaluate the current state of noninvasive fetal blood group genotyping. A pilot survey was originally performed at the 5th International Meeting on Cell‐Free DNA, cfDNA2023, in Copenhagen 2023. The survey was subsequently optimized, and new questions were added by a working group.

The final survey was sent out to all 63 national and international participants in the noninvasive fetal *RHD* genotyping 4268DK programme from the Danish Institute for External Quality Assurance for Laboratories in the health sector (DEKS) and to 29 members in the RCIBGT WP of the ISBT. In addition, the WP members were asked to disseminate the survey invitation to their network.

The survey was done in Microsoft Forms and the questions were divided into four sections: fetal *RHD* screening, fetal *RHD* genotyping for RhD immunized women, fetal genotyping of other targets than RhD (such as K, C, c, E and human platelet antigens [HPA]), and future collaboration. For the last section, the purpose was to evaluate both the interest for future collaborations in the community and to identify potential collaboration topics.

The survey was sent out twice with deadlines of 4th of June 2024 and 15th of September 2024. Data were compiled and discussed by the core author group.

## RESULTS

**FIGURE 1:**
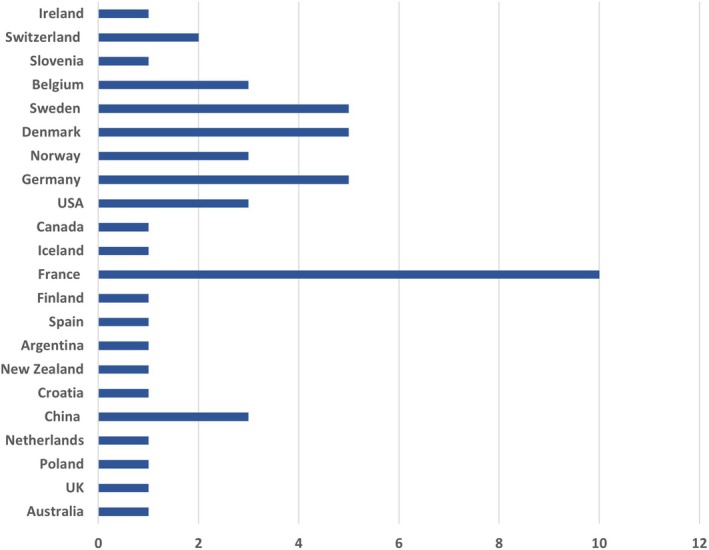
The country distribution of the survey responders (*n* = 52).

A total of 55 survey responses were recorded; 3 were omitted due to insufficient author/participant information, so in total, 52 responses were included in the evaluation. Each responder represented one laboratory (so that each laboratory was represented only once). Twenty‐two countries were represented, with 16 countries from Europe; see Figure 1. Results from Sections 1 to 3 of the survey are depicted in Tables 1, 2, 3.

**TABLE 1:** Section 1: Fetal *RHD* screening.

	*n*	%
Participants	52	
The countries represented are depicted in Figure 1		
Does your lab perform fetal *RHD* screening		
Yes	38	73
No	14	27
Is fetal *RHD* screening performed on request only?		
Request	5	13
Routinely	33	87
If you are not offering fetal *RHD* genotyping as screening, do you have any plans to introduce the service in the future?		
Yes	4	29
No	10	71
Is the fetal *RHD* screening performed as part of a nationwide routine service?		
Yes	27	71
No	11	29
In which year did you start fetal *RHD* screening as routine testing?		
2002	1	
2004	2	
2007	1	
2010	6	
2011	1	
2013	2	
2014	1	
2015	2	
2016	5	
2017	1	
2018	7	
2019	1	
2020	1	
2021	3	
2022	1	
2024	1	
How many samples do you test per year? See Figure 2a		
In which gestational week is the assay performed? See Figure 2b		
Which genotyping technique is used?		
Real‐time PCR	37	97
Digital PCR	1	3
Is an *in‐house* assay used?		
*In‐house*	26	68
Commercial	12	32
If *in‐house*, which exons are detected?		
Exon 5 and 7	11	
Exon 5 and 10	4	
Exon 7 and 10	5	
Exon 5, 7 and 10	4	
Exon 4, 5 and 10	2	
If commercial, which kit is used?		
Devyser	3	
FetoGnost® Kit	2	
Free DNA Fetal Kit® RhD	7	
What is your maximum allowed sample age (in days) before? DNA extraction is performed or freezing of the plasma?		
1 day	1	
2 days	4	
3 days	3	
4 days	1	
5 days	16	
6 days	3	
7 days	7	
8 days	2	
EDTA 3 days, cfDNA‐tube 4 days	1	
How is DNA extraction performed?		
Manual method	6	16
Automated method	32	84
Is a DNA extraction control performed?		
Yes	30	79
No	8	21
Please specify the extent of automation in your fetal *RHD* screening setup		
All manual steps	5	13
Mostly manual steps	6	16
Mostly automated steps	21	55
Fully automated	6	16
How is the result of the assay reported?		
Only the *RHD* result	5	13
*RHD* result and recommendations regarding prophylaxis	33	87
How is the finding of a maternal *RHD* allele managed?		
Prophylaxis is recommended (no additional maternal analysis)	15	40
Additional serology is performed on the maternal sample	4	10
Additional serology and genetic analysis are performed on the maternal sample	9	24
Additional genetic analysis is performed on the maternal sample	3	8
Other answers (see Results)	7	18
How is inconclusive result managed?		
Repeat on same sample	10	26
Request new sample	10	26
Recommend prophylaxis	5	13
All above (depending on context)	13	34
Is the result from *RHD* screen confirmed by cord blood typing of the newborn?		
Yes	13	34
No	19	50
Other (different policies)	6	16
Do you participate in any external quality control programmes?		
Yes	34	90
No	4	10
Which quality assessment service are you using?		
Asqualab	7	
Equalis	4	
DEKS	18	
DEKS and Equalis	1	
DEKS and Norwegian national EQA	2	
DEKS, Equalis and Norwegian national EQA	1	
DEKS and GenQA	1	
Are you planning to change your current fetal *RHD* screening method?		
From *in‐house* to commercial	7	
From real‐time PCR to NGS	‐	
From real‐time PCR to digital PCR	‐	
No	26	
Upgrading *in‐house* test to IVDR	1	
Not decided (but probably to commercial)	1	
Not decided (to digital)	1	
Recently changed from RT‐PCR to digital	1	
If required by IVDR, from *in‐house* to commercial	1	

Abbreviations: cfDNA, cell‐free DNA; EQA, external quality assurance; IVDR, In Vitro Medical Devices Regulation; NGS, next generation sequencing.

**TABLE 2:** Section 2: Fetal *RHD* genotyping for RhD immunized women.

	*n*	%
Is the assay used for fetal *RHD* screening also used for RhD immunized women?		
Yes	30	79
No	8	21
If no, what method is used?		
Fetal genotyping using ddPCR	1	
*In‐house* method for RHD exon 5, 7. Also includes SRY and CCR5	1	
Different RT‐PCR method	1	
PCR‐SSP combined with PCR‐RFLP	1	
Do not test RhD immunized women	4	
Are you using a fetal DNA marker?		
Yes	8	21
No	30	79
If not, are you checking negative results on a second sample?		
Yes	19	63
No	11	37

Abbreviations: ddPCR, droplet digital PCR; PCR‐RFLP, polymerase chain reaction—restriction fragment length polymorphis; PCR‐SSP, polymerase chain reaction with sequence‐specific primers; RT‐PCR, real‐time PCR.

**TABLE 3:** Section 3: Fetal genotyping of other targets (other than *RHD*).

	*n*	%
Do you test for non‐*RHD* targets?		
Yes	20	38
No	32	62
Which targets?		
FY*01/02, RHCE*c, E, C, KEL*01.01, ABO, JK, GYPA*01/02, GYPB*01/02, GYP*Mur, HPA‐1a/b, HPA‐5a/b, GAPDH, Maize DNA in plasma sample, *SRY, CCR5*, commercial process control, SOD		
Which genotyping technique(s) do you use?		
Real‐time PCR	15	
Digital PCR	2	
NGS	2	
Only serology	1	
Which sample tubes do you use?		
EDTA	19	95
Blood collection tube that stabilizes cell‐free DNA	1	5
What is your maximum allowed sample age (in days) before DNA extraction is performed or freezing of the plasma?		
2 days	6	
3 days	1	
5 days	6	
6 days	1	
7 days	2	
8 days	1	
No threshold	1	
Depending on collection tube; cfDNA tubes: 4 days, EDTA tubes: 3 days	1	
Not applicable	1	
Are you using a fetal DNA marker?		
Yes	8	40
No	12	60
If not, are you checking negative results on a second sample?		
Yes	8	
No	3	
Sometimes	1	
How many samples do you test per year (immunized women, non‐*RHD* targets)? See Figure 2c		
How many years of experience does your lab have with fetal genotyping? See Figure 2d		

Abbreviation: cfDNA, cell‐free DNA; NGS, next generation sequencing; SOD, superoxide dismutase.

### Fetal *RHD* screening

The questions and answers on fetal *RHD* screening are presented in Table 1. Of the 52 survey participants, 38 (73%) perform fetal *RHD* screening, with 27/38 running as routine, nationwide programmes. The technique applied is almost exclusively real‐time PCR, where 68% use *in‐house* methods (with different combinations of *RHD* exons 4, 5, 7 and 10) and 32% use commercially available kits. Samples are tested between 8 and 27 gestational weeks (GW), with most laboratories testing either around 10–12 GW or 24–27 GW. The number of days allowed for blood sample transportation spans diversely from 1 to 9 days before processing and DNA extraction. cffDNA is extracted from plasma mainly by automated DNA extraction. The degree of overall automation was reported as high. Most laboratories (87%) report the fetal *RHD* result in conjunction with recommendations for prophylaxis. Maternal *RHD* positive alleles and inconclusive results are dealt with diversely. In addition to the answers presented in Table 1, answers on handling maternally *RHD* positive alleles also included sporadic additional genetic testing, recommendation to base postnatal prophylaxis on cord blood testing (for such cases) and recommending antenatal prophylaxis except for women positive for Weak D types 1, 2 and 3. Inconclusive results were handled differently, with either repeat testing or recommending prophylaxis. Nineteen of thirty‐eight reported that they do not confirm the results from their screening setup by cord blood testing. Approximately 90% of the laboratories participate in external quality assurance, following either one or more programmes.

The majority of laboratories (26/38) have no plans for changing their setup, of which 12 use commercial kits and 14 use *in‐house* assays. Seven laboratories are planning to change from *in‐house* assays to commercial kits, and one laboratory is in the process of upgrading its assay to comply with In Vitro Medical Devices Regulation (IVDR).

### Fetal *RHD* genotyping for RhD immunized women

The questions and answers on fetal *RHD* genotyping for RhD immunized women are presented in Table 2. Of those laboratories running fetal *RHD* screening, 79% reported that they use the same analysis for RhD immunized women; otherwise, a different real‐time PCR or different techniques are used, including droplet digital PCR (ddPCR) and polymerase chain reaction with sequence‐specific primers (PCR‐SSP) combined with polymerase chain reaction—restriction fragment length polymorphism (PCR‐RFLP). Twenty‐one percent use a fetal marker, and of those laboratories not using a fetal marker, 63% retest their *RHD* negative results with a second sample.

### Fetal genotyping of targets other than RhD

The questions and answers of this section are presented in Table 3. Of the participating laboratories, 38% reported that they test for other fetal antigen targets than RhD. Several different targets, including C, c, E (*n* = 14) and K (*n* = 12) antigens, were the most commonly tested for (Table 3). The predominant technique used is real‐time PCR, but some laboratories use either DNA sequencing or ddPCR.

**FIGURE 2:**
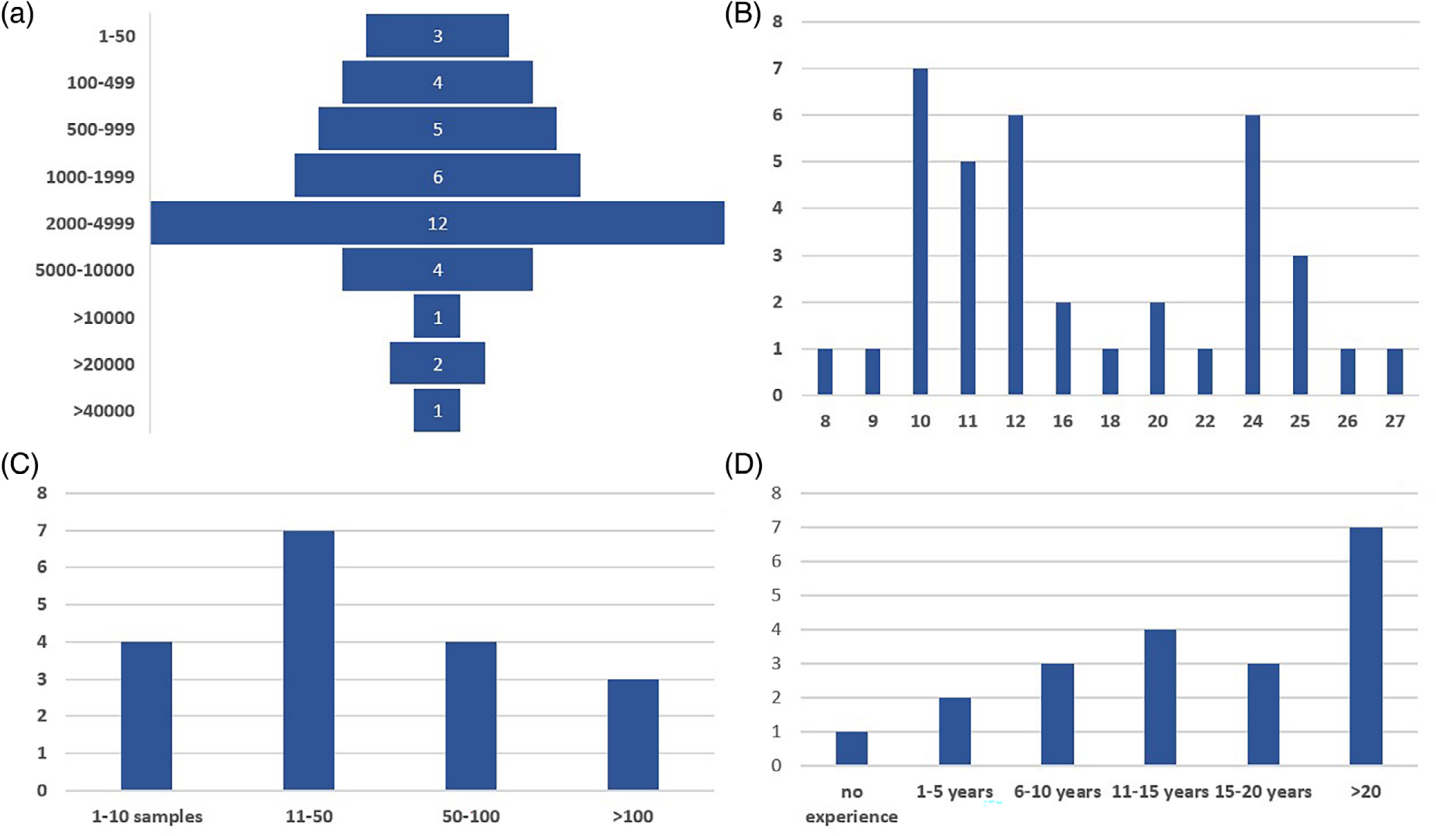
(a) How many fetal *RHD* screening samples do you test per year? The *y*‐axis shows the number of samples tested per year and the *x*‐axis shows the number of laboratories in each group. (b) In which gestational week is the fetal *RHD* screening performed? The *x*‐axis shows the week and the *y*‐axis shows the number of laboratories. (c) How many samples do you test per year for immunized women, non‐*RHD* target? The *x*‐axis shows the number of samples and the *y*‐axis shows the number of laboratories in each category. (d) How many years of experience does your laboratory have with fetal genotyping? The *x*‐axis shows the number of years and the *y*‐axis shows the number of laboratories in each category.

All laboratories with the exception of one use EDTA tubes for blood collection. The number of days allowed in transportation before processing was 2–8 days. Eight laboratories use a fetal marker, and 67% of those not using a fetal marker are retesting using a second sample. Eleven laboratories test <50 samples a year, and 7 laboratories test >50 samples a year (Figure 2c).

### Collaboration topics

In the final part of the survey, we asked for expression of interest in a future collaboration, and we asked for ideas for potential collaboration topics. Twenty‐seven responders answered that they were interested, 18 answered maybe and 7 answered no.

Potential collaboration topics provided by the responders included a wide range of ideas, from methodological and laboratory aspects to more strategic aspects related to new technologies, cost‐effectiveness and assay regulations by health care authorities.

## DISCUSSION

We investigated the status of clinical implementation of noninvasive fetal antigen testing. This was done via an online survey attended by 52 accepted/included responders. The main findings were that noninvasive fetal blood group genotyping is implemented in many countries, particularly in Europe. Almost 75% of the responders run routine fetal *RHD* screening, and most operate as national programmes. Testing in early pregnancy has become more common.

For immunized women, several targets of mainly blood groups are tested using various techniques. Although often considered a highly specialized diagnostic setup, almost 40% were testing for antigen targets other than RhD—indicating that this service has become common. Real‐time PCR is still the main method despite the arrival of novel techniques; this is likely due to its availability, price and robustness.

Interestingly, almost all laboratories use EDTA tubes for blood sampling, although benefits from using specialized tubes that limit the release of genomic DNA, such as Streck Cell‐Free DNA BCT*®*, are quite clear when testing cfDNA for antigen targets other than RhD [15].

For fetal *RHD* screening, accepted maximum sample transportation varied from 1 to 9 days. The international recommendation is preferably ≤5 days [16].

The final part of the survey was aimed at identifying topics for forthcoming collaborations. We will seek to categorize the suggested topics into separate groups and invite participation in those groups; this work will be carried out under the ISBT. Importantly, sharing knowledge and perhaps agreeing on test strategies as well as collaborating on solutions will advance this field and secure its future application in a clinical setting.

It is important that these noninvasive fetal antigen genotyping analyses are available for pregnant women, both as part of preventing immunization but also to take care of the already immunized women. With the novel, potential treatment with specially designed monoclonal antibodies, which block IgG recycling and placental transfer [17], early noninvasive fetal genotyping is vital to know if the treatment should start, especially for early‐onset severe anaemia.

Thus, noninvasive fetal antigen genotyping has vital roles, and widespread clinical implementation of these analyses is encouraged.

There are limitations to the study. It was difficult to reach out to all laboratories that perform fetal genotyping. We chose to contact participants partly via the quality assurance programme from DEKS. Consequently, the laboratories participating in the survey were biased towards having implemented programmes already. Although the survey was also for laboratories that did not run fetal genotyping, some laboratories decided not to participate because they did not run fetal genotyping, thus rendering a bias towards those who did.

There was a clear bias towards high‐income countries; consequently, the results are representative of high‐income countries only. There was also a higher number of laboratories participating from Europe compared with non‐European laboratories (with only 10 non‐European responders out of 52), rendering a bias towards Europe where these assays are more implemented into clinical routine [3, 9]. But as the frequency of true D negative women in Asia is low, there is less need for fetal *RHD* genotyping, and thus an underrepresentation of Asian laboratories in this survey was expected. Overall, the responders represent a substantial experience in this field and account for a total of approximately 170,000 samples tested yearly. Therefore, the results are highly interesting and reflect some of the challenges and differences of noninvasive fetal antigen genotyping. However, for future surveys we will optimize the survey invitation strategy, for example by exploiting the ISBT network more efficiently, so that we can target relevant laboratories more globally, including low‐ and middle‐income countries and countries with low RhD negative prevalence.

The survey questions were not comprehensive and may have included more detailed questions on, for example, *RHD* variants, investigating the current strategies used for testing in relation to fetal and/or maternal *RHD* variants. Similarly, we could have included questions pertaining to more clinical aspects, but a recent survey is covering these aspects [12]. In general, we chose to keep the survey short and simple to increase the chance for a high number of participations. Forthcoming surveys could address the aforementioned as well as other relevant aspects further.

## CONFLICT OF INTEREST STATEMENT

The authors declare no conflicts of interest.

## Data Availability

The data that support the findings of this study are available from the corresponding author upon reasonable request.

## APPENDIX A THE NONINVASIVE FETAL ANTIGEN GENOTYPING SURVEY GROUP

### A.1

Çiğdem Akalin Akkök, Department of Immunology and Transfusion Medicine, Oslo University Hospital, Norway. Aseel Alshamari, Department of Clinical Immunology and Transfusion, Örebro University Hospital, Örebro, Sweden. Ivana Babic, Croatian Institute of Transfusion Medicine, Department of Molecular Diagnostics, Zagreb, Croatia. Tonje Espeland Bagås, Department of Immunology and Transfusion Medicine, Haukeland University Hospital, Norway. Frédéric Bauer, Laboratoire centrale d'hématologie, CHUV, Lausanne, Switzerland. Jakob T. Bay, Department of Clinical Immunology, Zeeland University Hospital, Region Zeeland, Denmark. Rhita Bennis, CHU de Charleroi, Charleroi, Belgium. Gwen Clarke, University of Alberta, Canada. Jean‐Marc Costa, Laboratoire CERBA, Molecular Genetic Department, Frépillon, France. Carlos Cotorruelo, Laboratory of Immunohematology, National University of Rosario, Argentina. Christoffer D. Dellgren, Department of Clinical Immunology, Odense University Hospital, Odense, Denmark. Andrea Doescher, R&D, German Red Cross Blood Transfusion Service NSTOB, Oldenburg, Germany. Tadeja Dovc Drnovsek, Department of Immunohaematology, Blood Transfusion Centre of Slovenia, Ljubljana, Slovenia. Ducreux Stéphanie, LBM ALPIGENE, Lyon, France. Badrdine El Masmouhi, EFS, Paris, France. Willy Albert Flegel, Department of Transfusion Medicine, NIH Clinical Center, National Institutes of Health, Bethesda, MD, USA. Martine Florent, Hospital Avicenne EFS (Etablissement français du sang), Bobigny, France. Gatouillat Grégory, Lab. Immunology, CHU de Reims, France. Emmanuelle Guinchard, Laboratory of Erythrocyte‐Immunohematology and blood transfusion, Lyon‐GHE, France. Sys Hasslund, Department of Clinical Immunology, Aarhus University Hospital, Aarhus, Denmark. Christine Henny, Interregional Blood Transfusion SRC, Bern, Switzerland. Kaimo Hirv, MVZ Martinsried, Medicover Genetics, Germany. Camilla Holmertz, Department of Clinical Immunology and Transfusion medicine, Linköping, Sweden. Jayne Houghton, Royal Devon University Healthcare NHS Foundation Trust, Exeter, UK. Jauffret Vincent, Cytogen, Saint‐Herblain, France. Yanli Ji, Guangzhou Blood Center, Guangzhou, China. Hou Jue, Chengdu Blood Center, Chengdu, China. Elisa Kaivanto, Finnish Red Cross Blood Service, Finland. Margaret Keller, American Red Cross, Philadelphia PA, USA. Patrick S. L. Kwan, Clinical Development, New Zealand Blood Service, Auckland, New Zealand. Annie Levy, APHM Assistance Publique Hopitaux de Marseille, Marseille, France. Shuang Liang, Institute of Transfusion Medicine, Shenzhen Blood Center, Shenzhen, People's Republic of China. Yew‐Wah Liew, Red Cell Reference Laboratory, Australian Lifeblood, Brisbane, Australia. Loosen Claire, Centre Hospitalier EpiCURA, ATH—HORNU, Belgium. Agnès Mailloux, CNRHP‐AP‐HP Hôpital Saint‐Antoine, PARIS, France. Jean‐Marc Minon, Laboratory of Immunohematology and Blood Transfusion, Liege, Belgium. Kenneth J. Moise Jr, Dell Medical School‐UT Austin, Austin, Texas, USA. Núria Nogués, Immunohematology Laboratory, Banc de Sang i Teixits, Barcelona, Spain. Magnus Nordström, Department of Clinical Immunology and Transfusion, Umeå, Sweden. Agnieszka Orzińska, Institute of Haematology and Transfusion Medicine, Warsaw, Poland. Armin Pahl, LADR Central Laboratory Dr. Kramer & Collegues, Geesthacht, Germany. Cecilia Pardi, Department of Clinical Immunology and Transfusion, Sahlgrenska University Hospital, Gothenburg, Sweden. Pascal Pedini, French Blood Center, Marseille, France. Helen Ryan, Blood Group Genetics Laboratory, Irish Blood Transfusion Service, Dublin, Ireland. Janine‐Nicole Sorrentino, Amedes Medizinische Dienstleistungen GmbH, Hannover, Germany. Rudi Steffensen, Department of Clinical Immunology, Aalborg University Hospital, Aalborg, Denmark. Ingvild H. Sørvoll, University Hospital North, Tromsø, Norway. Jenny Bjork Thorsteinsdottir, The Blood Bank, Landspitali University Hospital, Iceland. Sandra Wienzek‐Lischka, Institute for Clinical Immunology, Transfusion Medicine and Haemostaseology, Justus‐Liebig‐University, Giessen, Germany.
